# Exact solution of the 1D Dirac equation for a pseudoscalar interaction potential with the inverse-square-root variation law

**DOI:** 10.1038/s41598-023-40604-9

**Published:** 2023-08-18

**Authors:** A. M. Ishkhanyan, V. P. Krainov

**Affiliations:** 1 Institute for Physical Research, 0204 Ashtarak, Armenia; 2 Moscow Institute of Physics and Technology, Dolgoprudny, Russia 141701

**Keywords:** Astronomy and astrophysics, Condensed-matter physics, Nuclear physics, Particle physics, Quantum physics

## Abstract

We present the exact solution of the one-dimensional stationary Dirac equation for the pseudoscalar interaction potential, which consists of a constant and a term that varies in accordance with the inverse-square-root law. The general solution of the problem is written in terms of irreducible linear combinations of two Kummer confluent hypergeometric functions and two Hermite functions with non-integer indices. Depending on the value of the indicated constant, the effective potential for the Schrödinger-type equation to which the problem is reduced can form a barrier or well. This well can support an infinite number of bound states. We derive the exact equation for the energy spectrum and construct a rather accurate approximation for the energies of bound states. The Maslov index involved turns out to be non-trivial; it depends on the parameters of the potential.

## Introduction

The Dirac equation is a relativistic wave equation that models the behavior of spin-1/2 particles in quantum mechanics and quantum field theory^1–3^. It is a generalization of the Schrödinger equation that takes into account the effects of special relativity. The Dirac equation has been used to study a wide variety of physical systems, including electrons, protons, neutrons, and quarks^1–3^. However, finding exact solutions of the Dirac equation for non-trivial interaction potentials is a challenging task, as it presents a rather complicated mathematical object.

One class of potentials that has attracted considerable attention in recent years is the pseudoscalar interaction potential^4–11^, which has been shown to be related to supersymmetry and integrability^12–15^, which are both important concepts in quantum mechanics. In this paper, we present the exact solution of the one-dimensional stationary Dirac equation for a pseudoscalar interaction potential, which is a combination of a constant and a term that varies in accordance with the inverse-square-root law. This potential is an *exactly* solvable potential because both of its parameters can be varied independently.

The general solution of the problem is written in terms of irreducible linear combinations of two Kummer confluent hypergeometric functions and two Hermite functions with non-integer indices. The effective potential for the Schrödinger-like equation, to which the problem is reduced, can be a repulsive potential or a well, depending on the value of the indicated constant. In the latter case, the potential supports infinitely many bound states, which are located in two energy intervals separated by the gap $( - mc^{2} ,mc^{2} )$. We derive the exact equation for the energy spectrum and construct a rather accurate approximation for the energies of bound states. The Maslov index^16^ (see also^17,18^) involved turns out to be non-trivial, unlike the case of a combined vector-scalar interaction with the same potential form^19^.

The paper is organized as follows. In Section "Potential", we review the one-dimensional Dirac equation with the pseudoscalar interaction potential under consideration, and the reduction of the equation to a single second-order differential equation. In Section "General solution", we present the exact general solution of the Dirac equation. In Section "Bound states", we derive the exact equation for the energy spectrum and construct a rather accurate approximation for it, which we compare to the exact numerical result. In Section "Discussion", we discuss the results of the paper and their implications. Since the applications of the pseudoscalar interaction potential extend to various areas of physics, we hope that this paper will be of interest to researchers in the fields of quantum mechanics, particle physics, and condensed matter physics.

## Potential

We consider a spin-1/2 Dirac fermion of rest mass $m$ and energy $E$ in the field of a pseudoscalar interaction potential given as

1 $$W(x) = W_{0} + \frac{{W_{1} }}{\sqrt x },$$

where $W_{0}$ and $W_{1}$ are arbitrary constants and $x$ is a space coordinate. The stationary one-dimensional Dirac equation for such an interaction can be written as

2 $$E\sigma_{0} \psi = \left( { - ic\hbar \sigma_{1} \frac{d}{dx} + W(x)\sigma_{2} + mc^{2} \sigma_{3} } \right)\psi ,$$

where $\psi = (\psi_{1} ,\psi_{2} )$ is the two-component wavefunction of the particle, $\sigma_{0}$ is the identity matrix, $\sigma_{1,2,3}$ are the Pauli matrices:

3 $$\sigma_{1} = \left( {\begin{array}{*{20}c} 0 & 1 \\ 1 & 0 \\ \end{array} } \right),\quad \sigma_{2} = \left( {\begin{array}{*{20}c} 0 & { - i} \\ i & 0 \\ \end{array} } \right),\quad \sigma_{3} = \left( {\begin{array}{*{20}c} 1 & 0 \\ 0 & { - 1} \\ \end{array} } \right),$$

$c$ is the speed of light, and $\hbar$ is the reduced Planck constant. In explicit form, the Dirac equation is written as

4 $$E\psi_{1} = - ic\hbar \frac{{d\psi_{2} }}{dx} - iW\psi_{2} + mc^{2} \psi_{1} ,$$

5 $$E\psi_{2} = - ic\hbar \frac{{d\psi_{1} }}{dx} + iW\psi_{1} - mc^{2} \psi_{2} .$$

Eliminating one of the components from this system, one can obtain a one-dimensional Schrödinger-like equation. For instance, resolving the second equation with respect to $\psi_{2}$:

6 $$\psi_{2} = \frac{{iW\psi_{1} - ic\hbar \psi^{\prime}_{1} }}{{E + mc^{2} }},$$

where the prime denotes differentiation, we obtain the following Schrödinger-type equation:

7 $$\frac{{d^{2} \psi_{1} }}{{dx^{2} }} + \frac{2m}{{\hbar^{2} }}\left( {E_{S} - V_{S} (x)} \right)\psi_{1} = 0,$$

where we have denoted

8 $$E_{S} = \frac{{E^{2} - m^{2} c^{4} }}{{2mc^{2} }}$$

and introduced the effective Schrödinger potential

9 $$V_{S} = \frac{{W^{2} + c\hbar W^{\prime}}}{{2mc^{2} }}.$$

Potential (1) is a version of the field configuration that was not discussed in previous studies of potentials with the same functional form, including terms that vary according to the law of inverse square root (see^19,20^). At first glance, the modification appears insignificant, with the only difference being the addition of a constant, $W_{0}$. However, this constant has a dramatic effect on the behavior of the system. Without $W_{0}$, the potential does not support bound states. However, for a *negative* value of $W_{0} W_{1}$, the potential supports infinitely many bound states, regardless of the values of the parameters $W_{0}$ and $W_{1}$. This is because in this case the potential becomes a long-range well that can trap particles from a long distance away. Importantly, for a definite-parity extension of potential (1) to the region $x < 0$, these observations hold for the entire $x$-axis.

**Figure 1:**
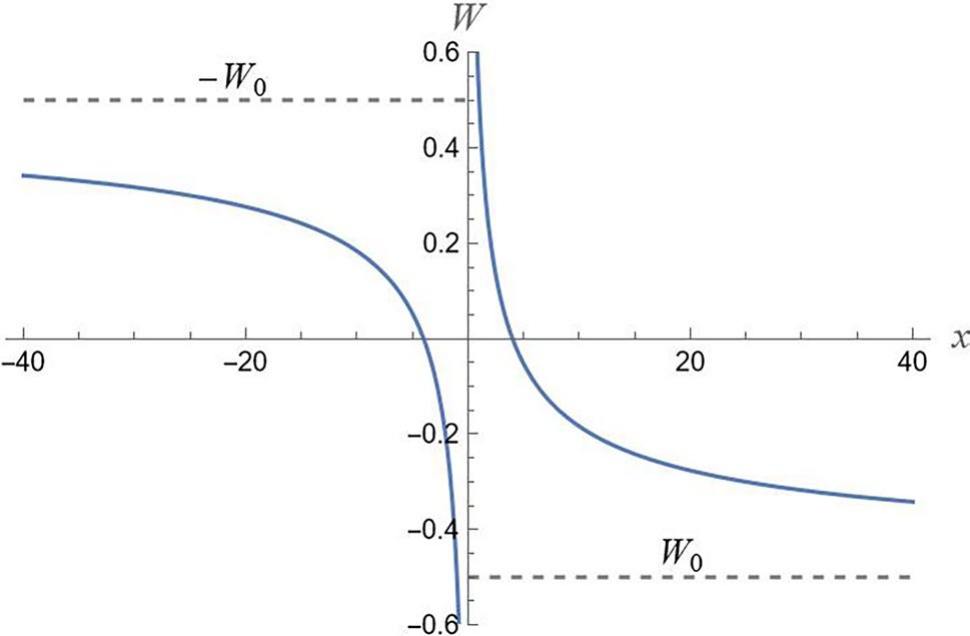
Odd-parity extension of potential (1); $\left( {W_{0} ,W_{1} } \right) = \left( { - 1/2,1} \right)$.

For an *odd-parity* extension (Fig. 1), we have

10 $$W = {\text{sgn}} (x)\left( {W_{0} + \frac{{W_{1} }}{{\sqrt {\left| x \right|} }}} \right)$$

and the effective Schrödinger potential is given as

11 $$V_{S} = \frac{1}{{2mc^{2} }}\left( {W_{0}^{2} + \frac{{2W_{1} W_{0} }}{{\sqrt {\left| x \right|} }} + \frac{{W_{1}^{2} }}{\left| x \right|} - \frac{{c\hbar W_{1} }}{{2\left| x \right|^{3/2} }}} \right).$$

**Figure 2:**
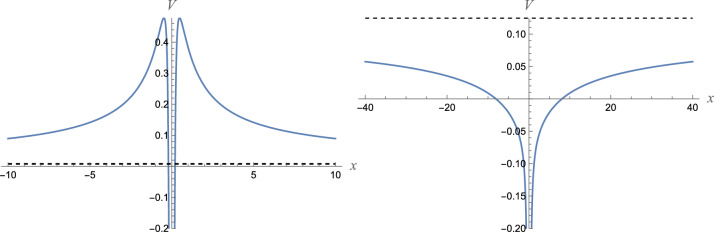
Schrödinger potential (11) for odd-parity extension (10) of potential (1). No bound states if $W_{0} = + 1/8 > 0$ (left panel) and infinitely many bound states if $W_{0} = - 1/2 < 0$ (right panel). Dashed line presents the limiting value $V( \pm \infty ) = W_{0}^{2} /(2mc^{2} )$. $(m,c,\hbar ,W_{1} ) = (1,1,1,1)$.

The form of this potential is shown in Fig. 2. As we can see, it is symmetric with respect to the origin. As the origin is approached, the potential diverges as $\left| x \right|^{ - 3/2}$. And as $x$ goes to infinity, the potential behaves as

12 $$\left. V \right|_{x \to \,\infty } \sim \frac{{W_{0}^{2} }}{{2mc^{2} }} + \frac{{W_{1} W_{0} }}{{mc^{2} }}\frac{1}{{\sqrt {\left| x \right|} }} + ....$$

Hence, it is a long-range potential. For $W_{1} W_{0} > 0$, the potential forms a barrier (Fig. 2, left panel), and for $W_{1} W_{0} < 0$, the potential forms a well (Fig. 2, right panel). In the latter case, the potential supports infinitely many bound states.

We conclude this section by noting that the ability of the background constant pseudoscalar potential to significantly impact the behavior of the system has been observed in several cases (see, for instance^21,22^).

## General solution

The effective Schrödinger potential (11) is a particular case of the *first* Exton potential (see Eq. (21) of^23^):

13 $$V_{E1} = V_{0} + \frac{{V_{1} }}{\sqrt x } + \frac{{8mV_{3}^{2} }}{{\hbar^{2} x}} + \frac{{V_{3} }}{{x^{3/2} }}.$$

Here, $V_{0,1,3}$ are arbitrary constants. This potential, in turn, is a specific case of one of the five biconfluent Heun potentials originally discussed by Lemieux and Bose^24^ (also see^25^):

14 $$V_{LB} = V_{0} + \frac{{V_{1} }}{\sqrt x } + \frac{{V_{2} }}{x} + \frac{{V_{3} }}{{x^{3/2} }}.$$

In general, the solution of the Schrödinger equation for this Lemieux-Bose potential is written in terms of the biconfluent Heun functions, which are advanced special functions^26–28^ that generalize the Kummer confluent hypergeometric function. This function is encountered in various branches of contemporary research, ranging from classical physics and quantum mechanics to general relativity and cosmology (see^29–34^ and references therein). Although the biconfluent Heun function is generally a complicated mathematical object, it can be simplified and expressed in terms of simpler mathematical functions, particularly confluent hypergeometric functions, for certain specifications of its potential. Many of these cases are obtained by terminating the expansion of the biconfluent Heun function in terms of the Hermite functions^35,36^. The first Exton potential (13) represents one such case. In this case, the solution is obtained by terminating the expansion at the second term. This simplified solution has been discussed in several papers^37–39^.

For $x > 0$, the effective Schrödinger potential given by Eq. (11) represents the first Exton potential with the specification

15 $$V_{0} = \frac{{W_{0}^{2} }}{{2mc^{2} }},\,\,\,\,\,\,V_{1} = \frac{{W_{0} W_{1} }}{{mc^{2} }},\,\,\,\,\,\,V_{3} = - \frac{{\hbar W_{1} }}{4mc}.$$

With this, the general solution of the Dirac equation can be written as^39^

16 $$\psi_{1} = e^{{ - b_{0} y + y^{2} /2}} \frac{du(y)}{{dy}},$$

17 $$u(y) = e^{{b_{0} y - y^{2} }} \left( {C_{1} H_{a - 1} \left( y \right) + C_{2} \cdot_{1} F_{1} \left( {\frac{1 - a}{2};\frac{1}{2};y^{2} } \right)} \right),$$

18 $$b_{0} = \frac{{2W_{1} \left( {2W_{0} + c\hbar \delta } \right)}}{{c^{2} \hbar^{2} \delta^{3/2} }},\quad y = \frac{{4W_{0} W_{1} }}{{c^{2} \hbar^{2} \delta^{3/2} }} + \sqrt {\delta x} ,$$

19 $$a = \frac{{8W_{1}^{2} (E^{2} - m^{2} c^{4} )}}{{c^{4} \hbar^{4} \delta^{3} }},\quad \delta = \pm \frac{2}{c\hbar }\sqrt {W_{0}^{2} + m^{2} c^{4} - E^{2} } ,$$

and $C_{1}$,$C_{2}$ are arbitrary constants. We note that both signs for $\delta$ are applicable; however, for definiteness, below we apply the plus sign. From Eqs. (16) and (17), we observe that each of the two independent fundamental solutions is given as an irreducible linear combination with non-constant coefficients of two Kummer hypergeometric functions or two Hermite functions with non-integer indices.

It can be verified that the wave function $\psi_{1}$ vanishes as $x$ goes to infinity only if $C_{2} = 0$. With this, the solution for $x > 0$ is simplified to

20 $$\psi_{{1{\text{R}}}} = C_{1} e^{{ - y^{2} /2}} \left( {2\left( {a - 1} \right)H_{a - 2} (y) + \left( {b_{0} - 2y} \right)H_{a - 1} (y)} \right).$$

To complete this section, we recall that the second component of the wave function is

21 $$\psi_{{2{\text{R}}}} = \frac{{iW\psi_{{1{\text{R}}}} - ic\hbar \psi^{\prime}_{{1{\text{R}}}} }}{{E + mc^{2} }}.$$

## Bound states

The possibility of bound states is determined by the boundary conditions at $x = 0$. Let the solution for $x < 0$ that vanishes at infinity be $\psi_{{\text{L}}} = (\psi_{{{\text{1L}}}} ,\psi_{{{\text{2L}}}} )$ and it be proportional to a constant, say $C_{3}$. Then the continuity condition of the wave function:

22 $$\left. {\psi_{{{\text{1L}}}} } \right|_{x \to - 0} = \left. {\psi_{{{\text{1R}}}} } \right|_{x \to + 0} ,$$

23 $$\left. {\psi_{{{\text{2L}}}} } \right|_{x \to - 0} = \left. {\psi_{{{\text{2R}}}} } \right|_{x \to + 0} ,$$

presents a set of two homogeneous linear equations with respect to the constants $C_{1}$ and $C_{3}$. For a non-trivial solution, the determinant of this system must be zero:

24 $$\left. {\psi_{{{\text{1L}}}} } \right|_{x \to - 0} \left. {\psi_{{{\text{2R}}}} } \right|_{x \to + 0} - \left. {\psi_{{{\text{1R}}}} } \right|_{x \to + 0} \left. {\psi_{{{\text{2L}}}} } \right|_{x \to - 0} = 0.$$

Since we consider the odd extension, when $W\left( { - x} \right) = - W\left( x \right)$, the Dirac equation is covariant under the parity transformation $x \to \,- x$, and the solution for the negative $x$-region can be written as $\psi_{{\text{L}}} (x) = C_{3} \left( {\psi_{{{\text{1R}}}} ( - x), - \psi_{{{\text{2R}}}} ( - x)} \right)$. As a result, Eq. (24) reduces to $\psi_{{1{\text{R}}}} \left( 0 \right)\psi_{{2{\text{R}}}} \left( 0 \right) = 0$, and we obtain two branches of bound states, generated by $\psi_{{1{\text{R}}}} \left( 0 \right) = 0$ or $\psi_{{2{\text{R}}}} \left( 0 \right) = 0$. Let us consider these cases separately.

### Bound states with $\psi_{{1{\text{R}}}} \left( 0 \right) = 0$

The equation for the bound states’ energy spectrum is

25 $$F\left( E \right) = 2\left( {a - 1} \right)H_{a - 2} (y_{0} ) + \left( {b_{0} - 2y_{0} } \right)H_{a - 1} (y_{0} ) = 0,\quad y_{0} = \frac{{4W_{0} W_{1} }}{{c^{2} \hbar^{2} \delta^{3/2} }}.$$

**Figure 3:**
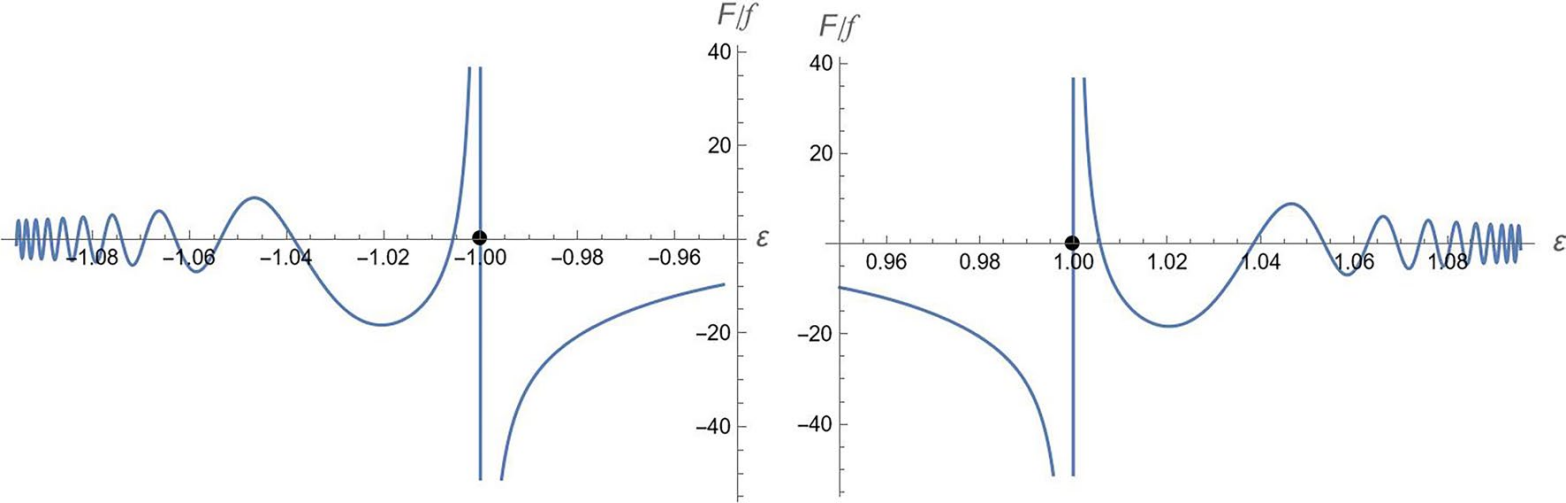
The behavior of function $F(E)$ of Eq. (25) in the negative (left panel) and positive (right panel) energy regions. The points show the position of $\varepsilon = \pm mc^{2}$. The roots are all located in the intervals $\left( { - \sqrt {m^{2} c^{4} + W_{0}^{2} } , - mc^{2} } \right)$ and $\left( {mc^{2} ,\sqrt {m^{2} c^{4} + W_{0}^{2} } } \right)$. $f = 2^{a/2} e^{{y_{0}^{2} /2}} \sqrt {(a + 1)!} ,\quad \left( {m,c,\hbar ,W_{1} ,W_{2} } \right) = \left( {1,1,1, - 1/2,1} \right)$.

The behavior of $F$ as a function of energy is shown in Fig. 3.

It can be shown that, provided $W_{0} W_{1} < 0$, the spectrum equation has infinitely many roots, all located in the intervals

26 $$E \in \left( { - \sqrt {m^{2} c^{4} + W_{0}^{2} } , - mc^{2} } \right)\quad and\quad E \in \left( {mc^{2} ,\sqrt {m^{2} c^{4} + W_{0}^{2} } } \right).$$

To construct an approximation for the spectrum, it is convenient to transform Eq. (25), using the recurrence relations between contiguous Hermite functions^40^, into the form

27 $$\frac{{2ay_{0} + b_{0} \left( {a + 1 - 2y_{0}^{2} } \right)}}{{b_{0} y_{0} - a}}H_{a + 1} (y_{0} ) + H_{a + 2} (y_{0} ) = 0.$$

The advantage of this form is that the argument $y_{0}$ of the involved Hermite functions here is such that it belongs to the *left transient region* $y_{0} \approx \sqrt {2\nu - 1}$, where $\nu = a + 1$ or $\nu = a + 2$. Then, using the Airy-function approximation of the Hermite function for this region^41^, we arrive at an approximation of this equation as

28 $$\sin \left( {\pi a - \frac{{A_{0} \left( {E_{0} - E} \right) + A_{1} \left( {E - mc^{2} } \right)}}{{E_{0} - mc^{2} }}} \right) = 0,$$

where

29 $$E_{0} = \sqrt {m^{2} c^{4} + W_{0}^{2} } ,$$

30 $$A_{0} = \frac{\pi }{2} - \tan^{ - 1} \left( {\frac{{Ai\left( {2^{1/3} 3^{1/6} \left( {\sqrt 2 u^{3/4} - \sqrt 3 } \right)} \right)}}{{Bi\left( {2^{1/3} 3^{1/6} \left( {\sqrt 2 u^{3/4} - \sqrt 3 } \right)} \right)}}} \right),$$

31 $$A_{1} = \frac{\pi }{2} - \tan^{ - 1} \left( {\frac{{Ai^{\prime } \left( u \right) - \sqrt u Ai\left( u \right)}}{{Bi^{\prime } \left( u \right) - \sqrt u Bi\left( u \right)}}} \right),$$

32 $${\text{and}}\quad u = \frac{{W_{1}^{4/3} }}{{\left( { - W_{0} c\hbar } \right)^{2/3} }}.$$

It is shown that $A_{0,1} < 1$ and for the solution of Eq. (29), $a > 1$. Furthermore, for large $a$, the energy $E$ is close to $E_{0}$: $E \approx E_{0}$. With these observations, for large a, we arrive at the approximate equation

33 $$\pi a - A_{1} = \pi n,\,\,\,\,\,n = 1,2,3,....$$

The dependence of $a$ on $E$ (see Eq. (19)) shows that this is a sextic polynomial equation in $E$ that is readily reduced to a cubic one. The real roots of the equation are given as

34 $$E_{n} = \pm \sqrt {m^{2} c^{4} + \left( {1 - e_{n} } \right)W_{0}^{2} } ,$$

where, using Cardano's formula for the cubic,

35 $$\begin{gathered} e_{n} = \frac{1}{3b} + \frac{{2^{1/3} \left( {6b - 1} \right)}}{{3b\left( {18b - 2 - 27b^{2} + 3\sqrt {b^{3} \left( {81b - 12} \right)} } \right)^{1/3} }} - \\ \frac{{2^{ - 1/3} }}{3b}\left( {18b - 2 - 27b^{2} + 3\sqrt {b^{3} \left( {81b - 12} \right)} } \right)^{1/3} \\ \end{gathered}$$

36 $${\text{and}}\quad b = \left( {\frac{{c\hbar W_{0} }}{{W_{1}^{2} }}} \right)^{2} \left( {n - 1 + \frac{{A_{1} }}{\pi }} \right)^{2} .$$

**Figure 4:**
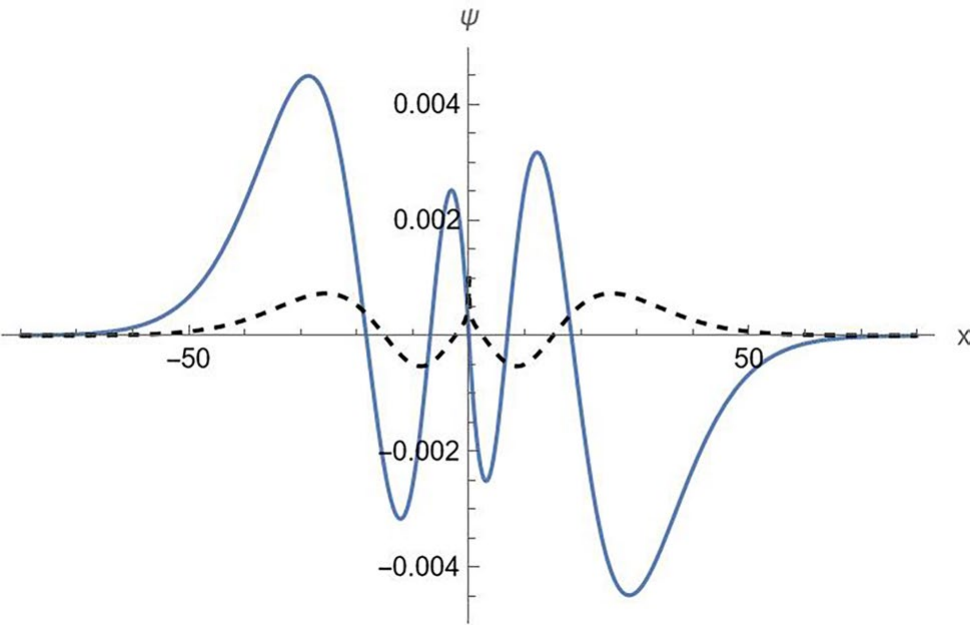
Normalized wave function with $\psi_{1} (0) = 0$ ($n = 3$, $E_{3} = 1.053641$). The solid line shows $\psi_{1}$ and the dashed line shows $\psi_{2} /i$. $\left( {m,c,\hbar ,W_{1} ,W_{2} } \right) = \left( {1,1,1, - 1/2,1} \right)$.

This is a fairly accurate approximation. It provides the spectrum with relative error of the order of $10^{ - 4}$ or less (see Table 1 for a comparison with the exact numerical result). The normalized wave function on the entire $x$-axis is shown in Fig. 4 ($n = 3$, positive energy branch). It can be observed that the wave function is anti-symmetric with respect to the origin, as expected, and that the derivative of $\psi_{2} (x)$ is discontinuous at the origin.

**Table 1:** Comparison of approximation (34) with the numerical solution of exact Eq. (27). $\left( {m,c,\hbar ,W_{1} ,W_{2} } \right) = \left( {1,1,1, - 1/2,1} \right)$.

$n$	1	2	3	4	5	6	7
$E_{n}$(exact)	1.005715	1.038481	1.053641	1.062871	1.069239	1.073967	1.077652
$E_{n}$(approx)	1.005603	1.038743	1.053759	1.062937	1.069281	1.073996	1.077672

### Bound states with $\psi_{{{\text{2R}}}} \left( 0 \right) = 0$

This time, the exact spectrum equation is written as

37 $$g\,H_{a + 1} (y_{0} ) + H_{a + 2} (y_{0} ) = 0,$$

38 $$g = \frac{{4\left( {2W_{1}^{2} - c^{2} \hbar^{2} \delta } \right)\left( {m^{2} c^{4} + W_{0}^{2} - E^{2} } \right) - 8W_{0} W_{1}^{2} \left( {c\hbar \delta - W_{0} } \right)}}{{W_{1} c^{2} \hbar^{2} \delta^{3/2} \left( {c\hbar \delta - 2W_{0} } \right)}},$$

**Figure 5:**
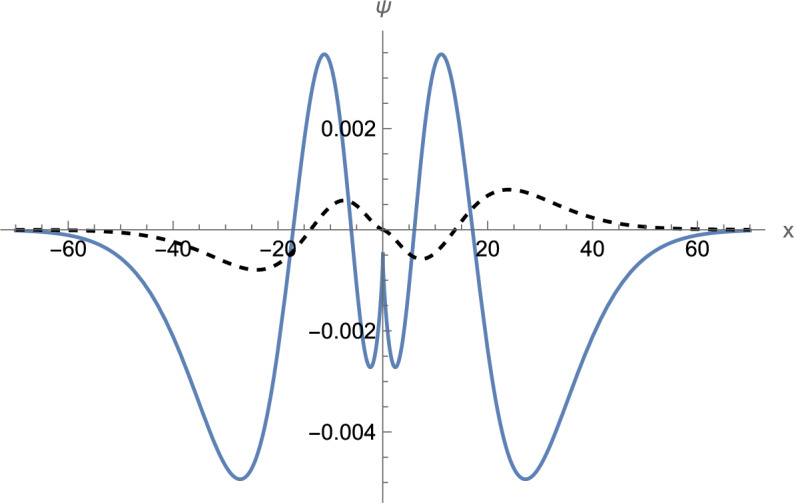
Normalized wave function with $\psi_{2} (0) = 0$ ($n = 3$, $E_{3} = 1.052395$). The solid line shows $\psi_{1}$ and the dashed line shows $\psi_{2} /i$. $\left( {m,c,\hbar ,W_{1} ,W_{2} } \right) = \left( {1,1,1, - 1/2,1} \right)$.

Acting now essentially in the same way as in the previous case, we arrive at the spectrum expressed by the same formulas (34)–(36), with the parameter $A_{1}$ given as

39 $$A_{1} = - \tan^{ - 1} \left( {\frac{{\sqrt u Ai\left( u \right) + Ai^{\prime } \left( u \right)}}{{\sqrt u Bi\left( u \right) + Bi^{\prime } \left( u \right)}}} \right),$$

where $u$ is given by Eq. (32). The obtained result again is a fairly good approximation as seen from Table 2. The normalized wave function on the entire $x$-axis is shown in Fig. 5 ($n = 3$, positive energy branch). It can be observed that this time the derivative of $\psi_{1} (x)$ is discontinuous at the origin.

**Table 2:** Comparison of approximation (34) with the numerical solution of exact Eq. (37). $\left( {m,c,\hbar ,W_{1} ,W_{2} } \right) = \left( {1,1,1, - 1/2,1} \right)$.

$n$	1	2	3	4	5	6	7
$E_{n}$(exact)	1.000000	1.036288	1.052395	1.062042	1.068638	1.073505	1.077283
$E_{n}$(approx)	1.000034	1.036279	1.052391	1.062040	1.068636	1.073504	1.077282

**Figure 6:**
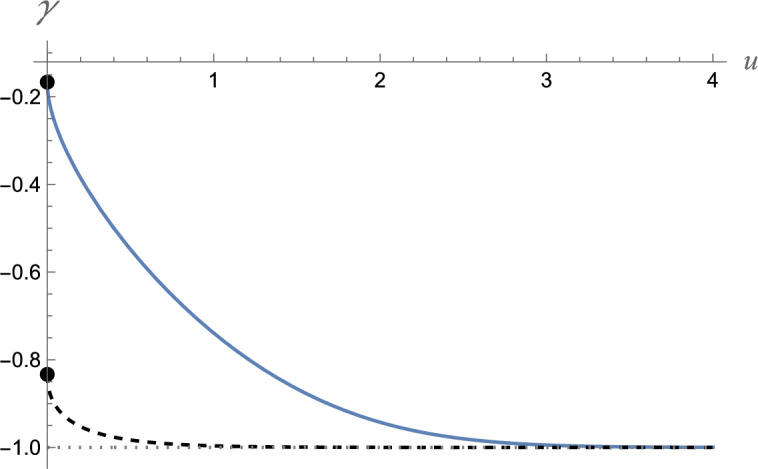
The dependence of the Maslov index on the parameter $u = W_{1}^{4/3} /\left( { - W_{0} c\hbar } \right)^{2/3}$. The solid line shows the index for the energy spectrum branch with $\psi_{1} (0) = 0$ and the dotted line stands for the branch with $\psi_{2} (0) = 0$. The points in the vertical axis indicate $- 1/6$ and $- 5/6$.

As Eq. (36) shows, the Maslov index is given as

40 $$\gamma = - 1 + \frac{{A_{1} }}{\pi },$$

where the parameter $A_{1}$ is different for the spectrum branches with $\psi_{1} (0) = 0$ and $\psi_{2} (0) = 0$. An interesting observation is that $A_{1}$ is not a constant but depends on the potential parameters $W_{0}$ and $W_{1}$. Figure 6 shows this dependence. As we can see, the Maslov index for the energy spectrum branch with $\psi_{1} (0) = 0$ starts from $- 1/6$, while that for the branch with $\psi_{2} (0) = 0$ starts from $- 5/6$ as $u = 0$. We note that both indices tend to $- 1$ as $u \to \infty$.

## Discussion

Thus, we have presented the exact solution of the one-dimensional stationary Dirac equation for a pseudoscalar potential consisting of an independently variable constant and a term that varies in accordance with the inverse square root law. Since the strength of the term that varies in accordance with the inverse square root law can also be independently varied, this is an exactly solvable potential. We have expressed the general solution of the problem as a linear combination with arbitrary coefficients of two fundamental solutions. Each of these fundamental solutions can be expressed as an irreducible linear combination of two functions of the hypergeometric class, namely Kummer hypergeometric functions or Hermite functions with non-integer indices.

A peculiarity of the potential we discussed is that the effective potential for the Schrödinger-like equation, to which the problem is reduced, changes its nature depending on the value of the involved constant term. With certain values, the effective potential is a barrier, and with others, it becomes a well, supporting infinitely many bound states.

We have derived the exact equation for the energy spectrum and have shown that the discrete energies of the bound states are located in two energy intervals separated by the gap $( - mc^{2} ,mc^{2} )$. We have constructed a rather accurate approximation for the energies of bound states and calculated the Maslov index of the spectrum. It turns out that this index, which is a constant addition to the quantum number $n$ numbering the bound states and does not vanish at $n \to \,\,\infty$, is rather nontrivial. In the Schrödinger case with a potential, which varies according to the inverse-square-root law, this index is known to be a constant equal to $- 1/6$^17,18^. On the contrary, in our case, the Maslov index turns out to be dependent on the potential parameters and may vary over a rather large interval.

The interaction we have discussed can serve as a model for studying relativistic quantum systems in one dimension, providing insight into confinement effects. The non-polynomial Hermite functions involved in the general solution, confinement behavior, and localization of wave functions near the origin contribute to a comprehensive understanding of relativistic quantum systems and hold promise for diverse applications in condensed matter physics and quantum technology. For example, this interaction can be used to model graphene nanoribbons^42,43^, Weyl and Dirac semimetals^44^, topological insulators and superconductors^45,46^, or quantum dots^47^.

## Data Availability

All data generated or analyzed during this study are included in this published article.
